# Omega-3 polyunsaturated fatty acid supplementation versus placebo on vascular health, glycaemic control, and metabolic parameters in people with type 1 diabetes: a randomised controlled preliminary trial

**DOI:** 10.1186/s12933-020-01094-5

**Published:** 2020-08-12

**Authors:** Lauren L. O’Mahoney, Gareth Dunseath, Rachel Churm, Mel Holmes, Christine Boesch, Antonios Stavropoulos-Kalinoglou, Ramzi A. Ajjan, Karen M. Birch, Nicolas M. Orsi, Georgia Mappa, Oliver J. Price, Matthew D. Campbell

**Affiliations:** 1 Carnegie School of Sport, Leeds Beckett University, Leeds, UK; 2 Diabetes Research Group, Swansea University Medical School, Swansea University, Swansea, UK; 3 Applied Sports, Technology, Exercise and Medicine (A-STEM) Research Centre, Swansea University, Swansea, UK; 4 School of Food Science and Nutrition, Faculty of Environment, University of Leeds, Leeds, LS2 9JT UK; 5 Leeds Institute of Cardiovascular and Metabolic Medicine, Faculty of Medicine and Health, University of Leeds, Leeds, UK; 6 School of Biomedical Sciences, University of Leeds, Leeds, UK; 7 Leeds Institute of Medical Research at St James’s, St James’s University Hospital, Leeds, UK

**Keywords:** Type 1 diabetes, Omega-3 polyunsaturated fatty acids, Vascular health, Glycaemic control

## Abstract

**Background:**

The role of omega-3 polyunsaturated fatty acids (n-3PUFA), and the potential impact of n-3PUFA supplementation, in the treatment and management of type 1 diabetes (T1D) remains unclear and controversial. Therefore, this study aimed to examine the efficacy of daily high-dose-bolus n-3PUFA supplementation on vascular health, glycaemic control, and metabolic parameters in subjects with T1D.

**Methods:**

Twenty-seven adults with T1D were recruited to a 6-month randomised, double-blind, placebo-controlled trial. Subjects received either 3.3 g/day of encapsulated n-3PUFA or encapsulated 3.0 g/day corn oil placebo (PLA) for 6-months, with follow-up at 9-months after 3-month washout. Erythrocyte fatty acid composition was determined via gas chromatography. Endpoints included inflammation-associated endothelial biomarkers (vascular cell adhesion molecule-1 [VCAM-1], intercellular adhesion molecule-1 [ICAM-1], E-selectin, P-selectin, pentraxin-3, vascular endothelial growth factor [VEGF]), and their mediator tumor necrosis factor alpha [TNFα] analysed via immunoassay, vascular structure (carotid intima-media thickness [CIMT]) and function (brachial artery flow mediated dilation [FMD]) determined via ultrasound technique, blood pressure, glycosylated haemoglobin (HbA1c), fasting plasma glucose (FPG), and postprandial metabolism.

**Results:**

Twenty subjects completed the trial in full. In the n-3PUFA group, the mean ± SD baseline n-3PUFA index of 4.93 ± 0.94% increased to 7.67 ± 1.86% (*P* < 0.001) after 3-months, and 8.29 ± 1.45% (*P* < 0.001) after 6-months. Total exposure to n-3PUFA over the 6-months (area under the curve) was 14.27 ± 3.05% per month under n-3PUFA, and 9.11 ± 2.74% per month under PLA (*P* < 0.001). VCAM-1, ICAM-1, E-selectin, P-selectin, pentraxin-3, VEGF, TNFα, CIMT, FMD, blood pressure, HbA1c, FPG, and postprandial metabolism did not differ between or within groups after treatment (*P* > 0.05).

**Conclusions:**

This study indicates that daily high-dose-bolus of n-3PUFA supplementation for 6-months does not improve vascular health, glucose homeostasis, or metabolic parameters in subjects with T1D. The findings from this preliminary RCT do not support the use of therapeutic n-3PUFA supplementation in the treatment and management of T1D and its associated complications.

*Trial Registration* ISRCTN, ISRCTN40811115. Registered 27 June 2017, http://www.isrctn.com/ISRCTN40811115.

## Background

The role of omega-3 polyunsaturated fatty acids (n-3PUFA), and the potential impact of n-3PUFA supplementation, in the treatment and management of diabetes remains unclear and controversial. Cross-sectional epidemiological studies suggest that inadequate n-3PUFA intake is associated with an increased risk of developing both type 2 diabetes (T2D) [1] and type 1 diabetes (T1D) [2]. However, a recent meta-analysis of randomised controlled trials (RCTs) concluded that n-3PUFA supplementation has little effect on the prevention of T2D in humans [3], and evidence for preventing T1D remains preliminary and limited to animal studies [4]. In murine models, research has shown that increased n-3PUFA intake reduces the risk of microvascular complications in vivo [5], and some studies [6, 7], although not all [8], have shown n-3PUFA supplementation improves biomarkers of vascular health and metabolic parameters in people with T2D [9, 10]. However, RCTs investigating n-3PUFA supplementation in T2D have failed to show reductions in major vascular events [11].

Dietary recommendations for people with and without diabetes currently advocate increased consumption of foods rich in n-3PUFA but do not recommend n-3PUFA supplementation [12]. However, the evidence underpinning these recommendations for people with diabetes is limited exclusively to people with T2D as very few RCTs have examined the impact of n-3PUFA supplementation on vascular or metabolic outcomes in T1D [13–18]. Further, most RCTs in T1D are confounded by short duration supplementation, low n-3PUFA dose, and failure to control for pretreatment fatty acid profile status. Therefore, the objective of this study was to investigate the efficacy of high-dose-bolus n-3PUFA supplementation on vascular health, glycaemic control, and metabolic parameters in adults with T1D.

## Methods

We performed a single-centre, parallel-group, double-blind, randomised, placebo-controlled trial in subjects with T1D, testing the efficacy of 6-months high-dose-bolus n-3PUFA supplementation on vascular health, glycaemic control, and metabolic parameters. Outcome assessments and safety outcomes were recorded at baseline, and after 3- and 6-months, with further assessment at 9-months following a subsequent 3-month washout (Additional file 1: Fig. S1). This trial was prospectively registered at the International Clinical Trials Registry Platform (ID: ISRCTN40811115), and ethical approval was granted by the local National Health Service Research Ethics Committee (17/NE/0244). In June 2018 a substantial ethics amendment was approved to increase the pool of available study participants. The amendment served to modify the inclusion criteria from “Not taking any prescribed medication other than insulin” to “Not taking any medication known to interact with n-3PUFA supplementation”. All subjects provided written informed consent before participating in the trial. Recruitment took place from September 2017–January 2019.

Twenty-seven adults aged 18–65 years with a diagnosis of T1D (glycosylated haemoglobin [HbA1c]: < 11% [97 mmol/mol]) > 2 years on enrolment and free from diabetes-related complications were eligible for inclusion. Exclusion criteria included any significant medical conditions including hepatic or haematological abnormalities, compromised glycaemic control (HbA1c > 11% (97 mmol/mol), current or previous pregnancy within the last 12-months or planning to become pregnant within 12-months, and taking any medication known to interact with n-3PUFA. All subjects were treated with a stable basal-bolus insulin regimen using either continuous subcutaneous insulin infusion (*n* = 9) or multiple daily injection (*n* = 11) therapy. Subjects following continuous subcutaneous insulin infusion therapy used insulins Aspart (*n* = 6) or Lispro (*n* = 3). For multiple daily injection therapy the basal component consisted of insulins Glargine (*n* = 5), Degludec (*n* = 2), Detemir (*n* = 3), or Isophane (*n* = 1) and the bolus component consisted of rapid-acting insulins Aspart (*n* = 8), Lispro (*n* = 2), or Glulisine (*n* = 1). Subjects were required to maintain their usual dietary habits and existing use of multivitamins throughout the duration of the study.

Following study enrolment, subjects were randomly assigned to treatment with n-3PUFA or placebo (PLA) in a 1:1 allocation ratio. Randomisation was conducted via the Minimization Program for allocating patients to clinical trials [19]; this algorithm ensures a balanced allocation of subjects across both groups for prognostic factors (i.e. age, BMI, diabetes duration). Randomization and safety monitoring as well as study drug labelling and dispensing were completed by staff not involved in recruitment or testing procedures. All subjects, clinical investigators, and outcome assessors were blinded to treatment allocation.

Subjects randomised to n-3PUFA supplementation ingested a daily high-dose-bolus of encapsulated 3.3 g/day (2.3 g EPA and 0.8 g DHA) (OmegaVia fish oil, OmegaVia, Calabasas, USA) over a 6-month period, whereas the placebo group ingested an encapsulated dose of 3.0 g/day corn oil (Pure corn oil, Mazola, Liverpool, UK). Capsules were aesthetically identical and dispensed in sealed pharmaceutical bottles. Monthly emails were sent individually to ensure compliance and monitor adverse effects. Subjects were instructed to retain packaging and informed that compliance would be objectively assessed through periodic blood analysis. On each laboratory visit, upper gastrointestinal symptoms (reflux, abdominal pain, and indigestion) were assessed via a self-administered validated questionnaire [20].

Subjects attended four separate but identical morning-time laboratory visits at baseline, 3-months, 6-months, and 9-months. Subjects refrained from caffeine, alcohol, and vigorous physical activity in the 48-h prior to each laboratory visit, in addition to adopting an overnight fast. During each visit, resting blood samples were obtained to analyse vascular and metabolic biomarkers. Vascular structure and function were assessed via ultrasound. Anthropometric measures (weight, body mass index, percentage body fat) were obtained via bioelectrical impedance analysis (SC-331S, Tanita, Amsterdam, Netherlands), and blood pressure was assessed via an automated oscillometric device (Intellisense HEM-907XL, Omron, Kyoto, Japan). In addition, subjects completed two subsequent mixed-meal tolerance tests to assess the potential impact of n-3PUFA supplementation on postprandial metabolism (see Additional file 1: Fig. S1 and Table S1).

On arrival to the laboratory, a 22-gauge cannula (Vasofix, B.Braun, Melsungen AG, Germany) was inserted into the antecubital vein of the non-dominant arm which was kept patent for periodic blood draws. Following initial measures (anthropometry and vascular assessment), subjects completed a meal-tolerance test (see Additional file 1: Table S1). At 4-h post-breakfast, subjects completed a second meal-tolerance test (see Additional file 1: Table S1) followed by a 4-h observation window. Subjects were instructed to consume each meal within a 20-min window and were permitted water intake ad libitum, for which quantity was recorded. Blood draws were made throughout the meal tolerance tests at 30-min intervals.

Venous blood samples were collected using 3 mL EDTA vacutainers and 6 mL lithium-heparin vacutainers (BD, New Jersey, USA). Fasting whole blood samples from each visit were analysed for HbA1c and erythrocyte fatty acid composition (Omegametrix GmbH laboratory, Planegg, Germany) via gas chromatography by methods described previously [21]; erythrocyte fatty acid composition was determined to establish baseline fatty acid status and was used as an objective measure of compliance to the intervention. The n-3PUFA index (O3I) was calculated as eicosapentaenoic acid plus docosahexaenoic acid. The remaining blood sample was centrifuged at 2700×*g* for 10 min at 4 °C and the resultant plasma was subsequently stored at − 80 °C for retrospective analysis of vascular biomarkers and metabolic parameters. A customised 7-plex human fluid-phase magnetic immunoassay (R&D Systems, Minneapolis, USA) was used for the simultaneous detection and quantification of vascular cell adhesion molecule-1 (VCAM-1), intercellular adhesion molecule-1 (ICAM-1), E-selectin, P-selectin, pentraxin-3, vascular endothelial growth factor (VEGF), and tumor necrosis factor alpha (TNFα) as per the manufacturer’s instructions. All vascular biomarker data were collected on a Luminex^®^ 200™ cytometer (Luminex, Texas, USA) and analysed using specialised software (Bio-Plex Manager 6.1, Bio-Rad, California, USA). In addition, fasting plasma glucose (FPG), triglycerides, cholesterol, and non-esterified fatty acids (NEFA) were quantified via an enzymatic colorimetric assay using a fully automated random-access analyser (Randox Daytona Plus, Randox Laboratories, UK). Further blood draws were made at 30-min intervals for the duration of the 8-h laboratory stay to capture postprandial glucose (PPGR) and triglyceride (PPTR) responses to each meal tolerance test; the trapezoidal rule was used to calculate individual areas under the plasma concentration curves (AUC); data is presented as sum AUC (0–8 h) for two consecutive mixed-meal tolerance tests. The intra-assay coefficient of variation was < 5% for all biochemical analyses.

Non-invasive high-resolution B-mode ultrasound imaging (GE Healthcare, Vivid I BT 12 EMEA, Illinois, USA) was performed using a GE 9L linear array ultrasound transducer probe (GE Healthcare, 12L-RS Linear Probe, Illinois, USA), to assess carotid-artery intima-media thickness (CIMT) and brachial artery flow mediated dilation (FMD) via previously described methods [22, 23]. Independently validated edge detection software (Vascular Research Tools 6, Medical Imaging Application, LLC, Iowa, USA) was used for offline analysis of all ultrasound images.

### Sample size and statistical analysis

Sample size for this study was estimated on the basis of prior work in T1D with shared similarities as well as published meal tolerance data [24–26]. Priori power calculations estimated a sample size of *n *= 16 in each arm would provide 80% power with α set at 0.05 to detect a small effect (*d *= 0.25) in the change variables for primary and secondary outcomes, namely: VCAM-1, E selectin, P-selectin, TNFα, pentraxin-3, ICAM-1, VEGF, HbA1c, triglycerides, PPGR, and PPGT [27]. On completion of the study, we used the realised sample size (20 subjects rather than 32) and standard error to calculate post hoc power for the observed estimates. Post-hoc power analysis on the attained sample size calculated the estimated power to be: > 99% for VCAM-1, E selectin, P-selectin, TNFα, pentraxin-3, FMD, CIMT, HbA1c, and triglycerides; 98% and 94% for PPTR and PPGR, respectively; 89% for ICAM-1; and 64% for VEGF.

Statistical analysis was performed using SPSS (Version 26, IBM, Illinois, USA). Primary analysis was per protocol and performed using a general linear mixed model repeated-measures analysis of variance. Secondary analysis was performed as intention to treat with last observation carried forward. The within-groups factor was time with treatment allocation as the between-groups factor. Pairwise comparisons were used to investigate significant interactions adjusted using a Bonferroni correction. Statistical models were further adjusted to include pretreatment fatty-acid exposure, and age, gender, age of diagnosis, duration of disease, and mode of insulin therapy as prognostic markers. Total exposure to n-3PUFA during the study period was calculated as the individual areas under the plasma concentration curves derived from the measurements at baseline and after 3-months, 6-months, and 9-months by using the trapezoidal rule. Statistical significance was set at *P *≤ 0.05. Data are presented mean ± SD.

## Results

### Study recruitment and follow-up

**Fig. 1:**
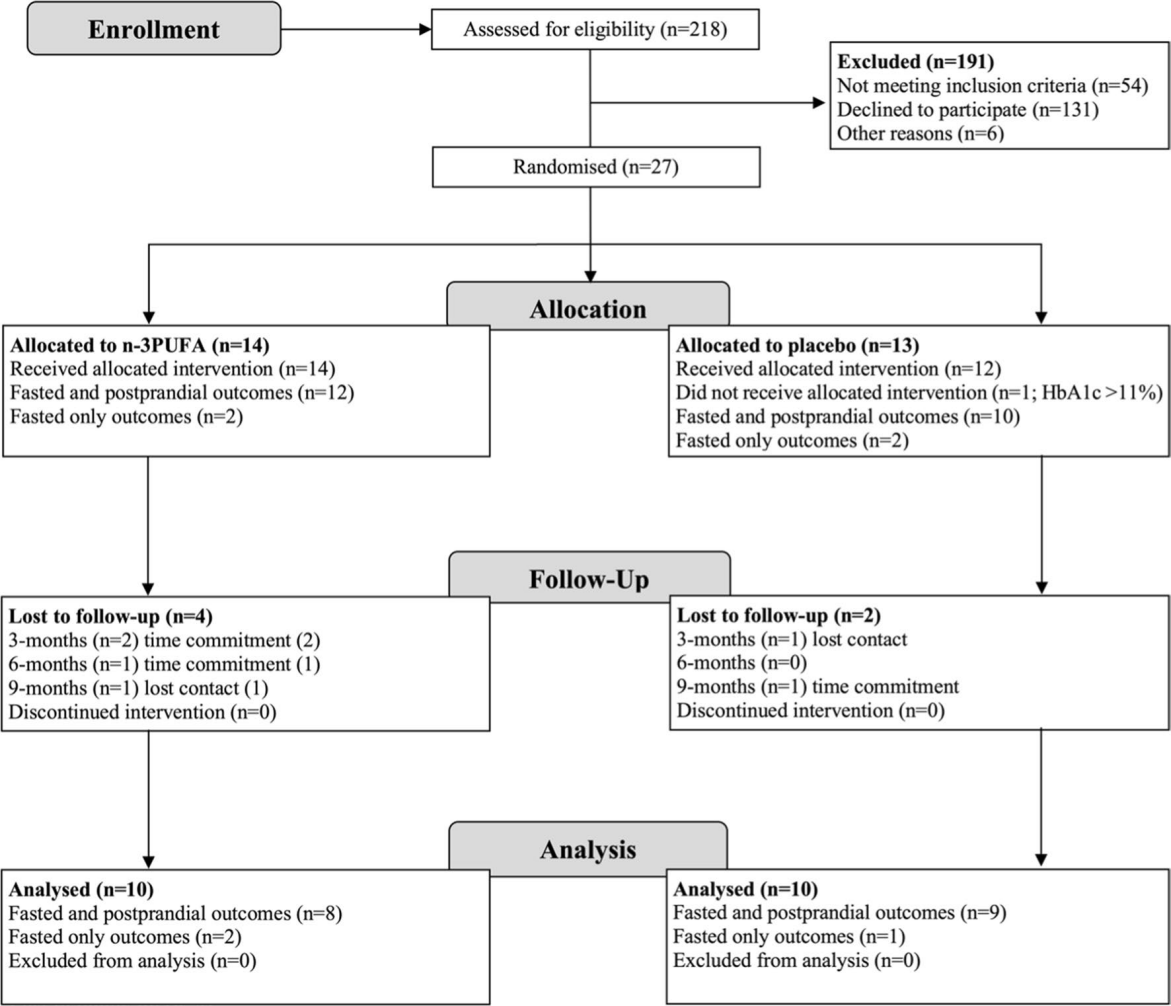
Subject enrolment, random assignment, and follow up reported in accordance with Consolidated Standards of Reporting Trials guidelines

We screened 218 subjects randomly allocating a total of 27 to treatment with n-3PUFA (*n *= 14) or PLA (*n *= 13) (Fig. 1). The main exclusion criteria were either significant medical conditions (42%) or pre-existing self-administration of n-3PUFA supplementation (25%). One subject was removed from the study shortly following baseline laboratory analysis due to elevated pretreatment HbA1c. Six subjects were lost to follow-up, and therefore 20 subjects completed the study in full (n-3PUFA, *n *= 10; PLA, *n *= 10). Subject demographic and pretreatment clinical characteristics are shown in Table 1.

**Table 1:** Pretreatment demographic and clinical characteristics of study subjects included in final analysis

	n-3PUFA	Placebo	*P* value
Total *n*	10	10	–
Gender			0.582^b^
Male	9	7	–
Female	1	3	–
Insulin therapy (*n*)			0.370^b^
Multiple daily injection	7	4	–
Basal insulin	4^G^; 2^D^; 1^DG^	1^D^; 1^DG^; 1^G^; 1^I^	
Bolus insulin	5^A^; 2^L^	3^A^; 1^GL^	
Continuous subcutaneous insulin infusion	3^A^	3^A^; 3^L^	–
Total daily insulin dose (IU)	41.3 ± 7.7	40.1 ± 9.5	0.776^a^
Medication	1 Atorvastatin 1 Lisinopril	1 Atorvastatin, Metformin, and Nefopam 1 Levothyroxine 1 Lisinopril and Pravastatin	1.000^b^
Age (y)	32 ± 12	36 ± 17	0.524^a^
Body mass index (kg/m^2^)	25.13 ± 3.43	28.14 ± 6.38	0.205^a^
Duration of diabetes (y)	15 ± 13	21 ± 12	0.284^a^
Age of diagnosis (y)	17 ± 8	16 ± 8	0.590^a^
Glycosylated haemoglobin (% [mmol/mol])	7.10 ± 3.20 [54.20 ± 11.95]	7.90 ± 3.40 [62.89 ± 13.81]	0.160^a^
Systolic blood pressure (mm/Hg)	127.3 ± 10.6	132.9 ± 11.7	0.275^a^
Diastolic blood pressure (mm/Hg)	78.0 ± 10.6	76.8 ± 6.2	0.751^a^
Triglycerides (mmol/L)	0.94 ± 0.58	1.04 ± 0.62	0.726^a^
Cholesterol (mmol/L)	4.25 ± 0.88	4.09 ± 0.45	0.637^a^
Omega-3 index (%)	4.93 ± 0.94	4.31 ± 1.15	0.206^a^

Data presented as mean ± SD. n-3PUFA compared to placebo. Independent samples t tests were performed on metric variables ^a^; Fisher exact tests performed on categorical variables ^b^. n-3PUFA, omega-3 polyunsaturated fatty acid. Omega-3 index calculated as eicosapentaenoic acid plus docosahexaenoic acid. A, Aspart; D, Detemir; DG, Degludec; G, Glargine; GL, Glulisine; I, Isophane; L, Lispro

### Outcomes

No significant differences were found between n-3PUFA and PLA groups in vascular, glycaemic, or metabolic endpoints (Table 2). The change in inflammatory-associated vascular endothelial proteins (VCAM-1, ICAM-1, VEGF, E-selectin, P-selectin, pentraxin-3) and TNFα measured as the difference between baseline and 6-months showed no significant differences between n-3PUFA and PLA (Table 2). Similarly, CIMT and FMD, as well as blood pressure remained unchanged in both n-3PUFA and PLA groups (*P *> 0.05; Table 2). The change in glycaemic control, measured as HbA1c, FPG, and PPGR, did not differ between groups; no within-group changes during treatment were found (*P *> 0.05; Table 2). Fasting cholesterol, NEFA, and triglyceride levels, as well as PPTR, remained unchanged and were not different within or between groups (*P *> 0.05; Table 2). Likewise, body weight, body mass index, and percentage body fat remained similar between the two treatment groups throughout the study (*P *> 0.05; Table 2). No changes in insulin requirements were observed with basal and bolus insulin doses remaining unchanged within each group. Bolus insulin administration was maintained for all mixed-meal tolerance tests across all four visits (n-3PUFA: 9.27 ± 3.00 IU; PLA: 8.04 ± 4.19 IU).

**Table 2:** 6-months high-dose-bolus n-3PUFA supplementation on vascular health, glycemic control, and metabolic parameters in T1D

Outcome	Pretreatment		3 months		6 months		9 months		*P* value
	n-3PUFA	Placebo	n-3PUFA	Placebo	n-3PUFA	Placebo	n-3PUFA	Placebo	
Vascular health									
Vascular cell adhesion molecule-1 (ng/mL)	823.83 ± 593.23	752.4 ± 631.97	865.58 ± 659.42	730.00 ± 610.72	848.12 ± 608.67	708.14 ± 691.22	843.17 ± 603.50	788.55 ± 603.53	0.833
Intercellular adhesion molecule-1 (ng/mL)	1225 ± 817	865 ± 205	1231 ± 845	945 ± 422	1221 ± 807	800 ± 170	1182 ± 796	831 ± 197	0.491
Vascular endothelial growth factor (pg/mL)	87.97 ± 45.03	75.06 ± 24.43	90.55 ± 47.56	87.54 ± 57.44	92.47 ± 52.52	63.74 ± 17.20	83.21 ± 45.49	71.18 ± 18.98	0.271
E-Selectin (ng/mL)	41.14 ± 21.79	45.45 ± 13.35	41.40 ± 21.93	44.64 ± 16.78	39.31 ± 20.74	41.53 ± 10.35	36.64 ± 18.76	44.54 ± 15.32	0.598
P-Selectin (ng/mL)	32.81 ± 12.51	37.73 ± 11.48	33.61 ± 14.05	37.70 ± 9.84	31.25 ± 11.57	35.17 ± 8.14	33.66 ± 14.06	35.29 ± 8.05	0.842
Pentraxin-3 (ng/mL)	3.80 ± 3.62	2.31 ± 1.25	3.89 ± 3.80	2.94 ± 2.88	3.77 ± 3.60	2.07 ± 0.98	3.71 ± 3.50	2.33 ± 1.31	0.529
Tumor necrosis factor alpha (pg/mL)	56.48 ± 41.77	59.25 ± 26.25	57.19 ± 43.15	61.57 ± 34.65	56.48 ± 41.76	55.67 ± 24.26	53.86 ± 38.26	57.80 ± 27.17	0.678
Far wall carotid intima-media thickness (mm)	0.61 ± 0.12	0.64 ± 0.09	0.59 ± 0.11	0.64 ± 0.09	0.60 ± 0.10	0.64 ± 0.09	0.60 ± 0.11	0.64 ± 0.09	0.216
Flow mediated dilation (%)	6.99 ± 1.45	7.42 ± 1.29	7.03 ± 1.48	7.24 ± 1.58	6.84 ± 1.61	7.25 ± 1.59	6.82 ± 1.36	7.26 ± 1.53	0.332
Systolic blood pressure (mm/Hg)	127 ± 11	133 ± 12	121 ± 11	127 ± 10	126 ± 6	123 ± 9	124 ± 12	124 ± 9	0.248
Diastolic blood pressure (mm/Hg)	78 ± 10	77 ± 6	69. ± 6	74 ± 7	69 ± 6	72 ± 7	68 ± 7	70 ± 5	0.466
Glycemic control									
Glycosylated haemoglobin (mmol/mol)	54.20 ± 11.95	62.89 ± 13.81	57.60 ± 11.70	61.22 ± 12.18	57.70 ± 13.63	64.33 ± 14.40	58.10 ± 12.24	62.00 ± 11.95	0.404
Fasting plasma glucose (mmol/L)^a^	11.51 ± 4.16	10.44 ± 4.02	12.40 ± 5.78	9.55 ± 4.93	12.25 ± 4.76	10.99 ± 4.28	13.12 ± 4.72	10.44 ± 3.11	0.798
PPGR (mmol/L/min)^c^	5423 ± 1223	5687 ± 3213	5979 ± 2042	5144 ± 2294	6030 ± 1856	5169 ± 2435	6883 ± 1975	5324 ± 2253	0.135
Metabolic parameters									
Triglycerides (mmol/L)^a^	1.02 ± 0.65	1.11 ± 0.63	0.98 ± 0.41	1.07 ± 0.55	0.92 ± 0.54	1.15 ± 0.49	1.06 ± 0.56	1.20 ± 0.69	0.858
PPTR (mmol/L/min)^c^	610.08 ± 331.62	662 ± 401	578 ± 209	600 ± 227	567 ± 243	676 ± 398	641 ± 173	669 ± 326	0.792
Cholesterol (mmol/L)^b^	4.23 ± 0.91	4.11 ± 0.47	4.22 ± 0.45	4.16 ± 0.59	5.01 ± 1.46	3.96 ± 0.71	4.80 ± 1.60	4.33 ± 1.44	0.432
Non-esterified fatty acids (mmol/L)^b^	0.37 ± 0.18	0.57 ± 0.36	0.48 ± 0.37	0.39 ± 0.16	0.38 ± 0.40	0.54 ± 0.32	0.52 ± 0.36	0.55 ± 0.29	0.254
Anthropometry									
Body weight (kg)	82.56 ± 15.34	80.22 ± 19.07	82.56 ± 14.30	80.83 ± 18.61	82.15 ± 15.47	80.96 ± 18.85	82.53 ± 14.96	80.56 ± 17.87	0.669
Body mass index (kg/m^2^)	25.13 ± 3.43	28.14 ± 6.38	25.20 ± 3.29	27.13 ± 4.78	25.10 ± 3.71	27.40 ± 5.10	25.19 ± 3.40	27.25 ± 4.44	0.552
Body Fat (%)	19.43 ± 6.86	26.47 ± 15.47	19.65 ± 6.73	25.09 ± 10.85	19.48 ± 7.49	25.83 ± 11.68	19.36 ± 7.50	24.68 ± 11.17	0.739

*n *= 10 in both groups unless stated otherwise. Values are presented as mean ± SD. Follow-up at 9-months was after 3-months washout. n-3PUFA, omega-3 polyunsaturated fatty acid; ^a^, *n *= (n-3PUFA: 9; PLA: 9); ^b^, *n *= (n-3PUFA: 6; PLA: 8); ^c^, *n *= (n-3PUFA: 6; PLA: 7); PPGR, postprandial glucose response; PPTR, postprandial triglyceride response. Postprandial responses presented as sum of the area under the curve (0–8 h) following two consecutive mixed-meal tolerance tests

### Erythrocyte fatty acid composition

In the n-3PUFA group, baseline O3I of 4.93 ± 0.94% increased to 7.67 ± 1.86% (*P* < 0.001) after 3-months, and 8.29 ± 1.45% (*P* < 0.001) after 6-months, before decreasing to 5.84 ± 0.22% (*P *= 0.005) at 9-months following washout. Total exposure to n-3PUFA over the 6-months (area under the curve) was 14.27 ± 3.05% per month and 9.11 ± 2.74% per month in the n-3PUFA and PLA groups, respectively (*P* < 0.001). Comprehensive erythrocyte fatty acid profiles for saturated, monounsaturated, and polyunsaturated fatty acids across both groups at all time points are presented in Additional file 1: Table S2.

Adjustment for pretreatment fatty acid composition, age, gender, age of T1D diagnosis, duration of diabetes, or mode of insulin therapy did not impact outcomes (Additional file 1: Table S3). Intention-to-treat analyses on the basis of last observation carried forward did not significantly alter outcomes (Additional file 1: Table S5).

### Safety

Overall, no important safety issues occurred during n-3PUFA supplementation. The mean severity of gastrointestinal side effects assessed did not significantly differ over time for either treatment condition. The reported severity of gastrointestinal symptoms changed from no symptoms to mild during the 6-month supplementation period in seven subjects (n-3PUFA: *n *= 2; PLA: *n *= 5), whereas symptoms changed from mild to moderate in five subjects (n-3PUFA: *n *= 3; PLA: *n *= 2).

## Discussion

In this RCT of n-3PUFA versus placebo in subjects with T1D, daily high-dose-bolus n-3PUFA supplementation for 6-months did not improve vascular health, glycaemic control, or metabolic parameters in subjects with T1D. These findings have important clinical implications concerning dietary recommendations and nutritional guidance for people with T1D. Specifically, our findings question the efficacy of n-3PUFA supplementation for the treatment and management of T1D and its associated complications. Our data support previous studies conducted in T2D patients [28–32] and provide new insight within the context of T1D [13–18].

This is the first study to comprehensively assess the impact of n-3PUFA supplementation on vascular health in subjects with T1D. We have previously shown that inflammation and vascular markers are raised in people with T1D, even in those with good metabolic control [26, 33]. The pretreatment inflammatory cytokine and endothelial cell adhesion molecule concentrations of patients in the present study, appear elevated compared to previously published data in T1D [26, 34]. Such differences may be due to differences in clinical characteristics (i.e. age, BMI, and diabetes duration) between study populations and indicates increased endothelial activation our patients. It is widely recognised that vascular adhesion molecules play an important pathophysiological role in atherosclerosis [35], and are upregulated early into the progression of microvascular complications in subjects with T1D [36]. n-3PUFAs have previous been shown to attenuate the expression of adhesion molecules and vascular inflammation in human endothelial cells in vitro [9, 10, 37]. Additionally, the metabolism of n-3PUFA: (i) modulates macrophage functions [38], (ii) produces bioactive metabolites which possess anti-inflammatory properties [39], and (iii) reduces atherogenesis and platelet aggregation [40]. A high n-6PUFA:n-3PUFA ratio has also been associated with increased systemic inflammation in T2D [41]. Therefore, it is plausible to speculate that similar improvements in vascular inflammation would be observed in people with T1D following n-3PUFA supplementation. Notably, however, no significant improvements were detected in vascular adhesion molecules or their mediator TNFα in the present study. In addition, we found no effect of n-3PUFA supplementation on CIMT, FMD, or blood pressure. Previous research investigating n-3PUFA supplementation in T2D has reported conflicting results regarding CIMT [32, 42] and blood pressure responses [6, 43].

Further, we report for the first time, the impact of n-3PUFA supplementation on postprandial metabolism. Postprandial metabolic handling is a strong and independent predictor of cardiovascular disease [44, 45] and a critical component of T1D self-management. We show that a daily high-dose-bolus of n-3PUFAs does not modulate postprandial glycemia or lipemia. Additionally, it was found n-3PUFA supplementation does not improve long-term glycaemic control or metabolic parameters. Prior research investigating the utility of n-3PUFAs in T1D has shown both improvements [18] and no change on glucose homeostasis [13, 14, 17]. The beneficial effects of n-3PUFAs on fasting triglycerides are well documented in T2D [46]. Although not statistically significant, we observed a numerical reduction in fasting triglyceride levels in the n-3PUFA arm at 3-months and 6-months that was restored following washout. In our study, changes in fasting triglycerides from 3 to 9 months in the PLA group may have masked a statistically significant effect in the n-3PUFA group. Although speculative, one potential mechanism for triglyceride reduction is decreased secretion of inflammatory cytokines, which reduces hepatic very low-density lipoprotein synthesis and accelerates chylomicron clearance [47, 48]. Considering our chosen inflammatory biomarkers, particularly TNFα, remained unchanged, this could, in part, explain why triglyceride levels in our participants remained statistically unchanged. Although, reductions in fasting triglyceride levels have been shown in response to n-3PUFAs independent of baseline triglyceride values, higher reductions in triglycerides are observed when baseline triglycerides are elevated [49–51]. Further, the majority of our subjects presented with relatively good glycaemic control (~ 7.1% [~ 54 mmol/mol]) with triglyceride levels < 1.0 mmol/L at study entry. Thus, we cannot exclude the possibility that n-3PUFA supplementation over a longer duration or in less well-controlled subjects (± comorbidities) may have nongenomic effects on glycaemic control, metabolic health or vascular health, that were not detectable in our cohort or by our study design. Whilst we administered n-3PUFA at a dose above the 2 g/day minimum suggested necessary to elicit beneficial cardiovascular effects [52], the current study focused on the additive effects of n-3PUFA to habitual dietary habits given the known poor adherence to chronic dietary changes [53]. However, research suggests that n-3PUFA supplementation alone is insufficient and needs to replace saturated fatty acid intake in order to modify CVD risk [54]. While increased n-3PUFA intake may reduce the risk of developing T1D [55], the current study questions the therapeutic utility and disease modifying potential of n-3PUFA in adults with T1D.

### Strengths and limitations

Although the strengths of this study include its double-blind randomised placebo-controlled design; long supplementation duration and daily high dose of n-3PUFA; assessment of legacy effects and follow-up after washout; objective assessment of compliance via erythrocyte gas chromatography; the inclusion of a broad and comprehensive assessment of outcomes using gold-standard measures; and control of pretreatment fatty acid composition and subject characteristics it is not without limitations. Firstly, the broad selection of subjects with different treatment regimens, duration of disease, and sex may have caused heterogeneity in the study sample and may have masked a positive effect. However, controlling for these potentially confounding factors in our statistical analysis did not change the outcome of study findings and increases generalizability. The obtained sample size was relatively small and the number of subjects lost to follow up was proportionately high. However, our sample size was deemed sufficient to yield adequate statistical power across outcomes, and is comparable to previously published RCTs assessing the effectiveness of n-3PUFA supplementation in adults with T1D [14, 18].

## Conclusions

This study indicates that daily high-dose-bolus n-3PUFA supplementation for 6-months does not improve vascular health, glucose homeostasis, or metabolic parameters in well controlled subjects with T1D without existing microvascular complications. Despite the lack of evidence to support clinical benefit, n-3PUFA supplementation is common in people living with T1D. However, the findings from this preliminary RCT do not support the use of therapeutic n-3PUFA supplementation in the treatment and management of T1D and its associated complications. Future research is needed to assess the effectiveness of other potential adjunct therapies that may act to modify the markedly elevated CVD risk amongst adults with T1D.

## Supplementary information

**Additional file 1: Figure S1.** Schematic representation of study design. n-3PUFA, omega-3 polyunsaturated fatty acid; = blood sampling; ultrasound imaging. Laboratory visits were scheduled at week 0, 12, 24, and 36. **Table S1.** Macronutrient composition of mixed-meal tolerance tests. **Table S2.** Erythrocyte fatty acid profiles in response to n-3PUFA supplementation or placebo in adults with type 1 diabetes. Mean ± SD. *n*=10 in each group. *, p<0.05; **, p<0.01; ***, p<0.001. n-3PUFA, omega-3 polyunsaturated fatty acids. All values are expressed as a percentage of total identified fatty acids after response factor correction. **Table S3.** Adjusted analysis for the effects of n-3PUFA supplementation on multiple health outcomes in adults with type 1 diabetes. **Table S4.** Pre-treatment demographic and clinical characteristics of intention-to-treat cohort. **Table S5.** Effect of omega-3 polyunsaturated fatty acids (n3PUFA) or placebo on cardiovascular and metabolic biomarkers, glycaemic parameters, measures of anthropometry, and vascular structure and function as per intention-to-treat analysis.

## Data Availability

The datasets used and/or analysed during the current study are available from the corresponding author on reasonable request.

## Abbreviations

CIMT: Carotid intima-media thickness
FPG: Fasting plasma glucose
FMD: Flow mediated dilation
HbA1c: Glycosylated haemoglobin
ICAM-1: Intercellular adhesion molecule-1
n-3PUFA: Omega-3 polyunsaturated fatty acid
NEFA: Non-esterified fatty acid
O3I: Omega-3 Index
PLA: Placebo
PPGR: Postprandial glucose response
PPTR: Postprandial triglyceride response
RCT: Randomised controlled trial
T1D: Type 1 diabetes
T2D: Type 2 diabetes
TNFα: Tumor necrosis factor alpha
VCAM-1: Vascular cell adhesion molecule-1
VEGF: Vascular endothelial growth factor

## References

[1] Brostow DP, Odegaard AO, Koh W-P, Duval S, Gross MD, Yuan J-M, Pereira MA (2011). Omega-3 fatty acids and incident type 2 diabetes: the Singapore Chinese Health Study. Am J Clin Nutr.

[2] Norris JM, Yin X, Lamb MM, Barriga K, Seifert J, Hoffman M, Orton HD, Baron AE, Clare-Salzler M, Chase HP (2007). Omega-3 polyunsaturated fatty acid intake and islet autoimmunity in children at increased risk for type 1 diabetes. JAMA.

[3] Brown TJ, Brainard J, Song F, Wang X, Abdelhamid A, Hooper L (2019). Omega-3, omega-6, and total dietary polyunsaturated fat for prevention and treatment of type 2 diabetes mellitus: systematic review and meta-analysis of randomised controlled trials. BMJ.

[4] Bi X, Li F, Liu S, Jin Y, Zhang X, Yang T, Dai Y, Li X, Zhao AZ (2017). ω-3 polyunsaturated fatty acids ameliorate type 1 diabetes and autoimmunity. J Clin Invest.

[5] Sapieha P, Chen J, Stahl A, Seaward MR, Favazza TL, Juan AM, Hatton CJ, Joyal JS, Krah NM, Dennison RJ (2012). Omega-3 polyunsaturated fatty acids preserve retinal function in type 2 diabetic mice. Nutr Diabetes.

[6] O’Mahoney LL, Matu J, Price OJ, Birch KM, Ajjan RA, Farrar D, Tapp R, West DJ, Deighton K, Campbell MD (2018). Omega-3 polyunsaturated fatty acids favourably modulate cardiometabolic biomarkers in type 2 diabetes: a meta-analysis and meta-regression of randomized controlled trials. Cardiovascular Diabetol.

[7] Chew EY (2017). Dietary intake of omega-3 fatty acids from fish and risk of diabetic retinopathy. JAMA.

[8] McCormick KG, Scorletti E, Bhatia L, Calder PC, Griffin MJ, Clough GF, Byrne CD (2015). Impact of high dose n-3 polyunsaturated fatty acid treatment on measures of microvascular function and vibration perception in non-alcoholic fatty liver disease: results from the randomised WELCOME trial. Diabetologia.

[9] Goua M, Mulgrew S, Frank J, Rees D, Sneddon AA, Wahle KW (2008). Regulation of adhesion molecule expression in human endothelial and smooth muscle cells by omega-3 fatty acids and conjugated linoleic acids: involvement of the transcription factor NF-kappaB?. Prostaglandins Leukot Essent Fatty Acids.

[10] Wang TM, Chen CJ, Lee TS, Chao HY, Wu WH, Hsieh SC, Sheu HH, Chiang AN (2011). Docosahexaenoic acid attenuates VCAM-1 expression and NF-kappaB activation in TNF-alpha-treated human aortic endothelial cells. J Nutr Biochem.

[11] Aung T, Halsey J, Kromhout D, Gerstein HC, Marchioli R, Tavazzi L, Geleijnse JM, Rauch B, Ness A, Galan P (2018). Associations of omega-3 fatty acid supplement use with cardiovascular disease risks: meta-analysis of 10 trials involving 77917 individuals. JAMA Cardiol.

[12] National Institute for Health and Care Excellence: Cardiovascular disease: risk assessment and reduction, including lipid modification [http://www.nice.org.uk/Guidance/CG181]. Accessed 21 Feb 2020.

[13] Mori TA, Vandongen R, Masarei JR, Rouse IL, Dunbar D (1991). Comparison of diets supplemented with fish oil or olive oil on plasma lipoproteins in insulin-dependent diabetics. Metabolism.

[14] Mori TA, Vandongen R, Masarei JR (1990). Fish oil-induced changes in apolipoproteins in IDDM subjects. Diabetes Care.

[15] Jensen T, Stender S, Goldstein K, Holmer G, Deckert T (1989). Partial normalization by dietary cod-liver oil of increased microvascular albumin leakage in patients with insulin-dependent diabetes and albuminuria. N Engl J Med.

[16] Hamazaki T, Takazakura E, Osawa K, Urakaze M, Yano S (1990). Reduction in microalbuminuria in diabetics by eicosapentaenoic acid ethyl ester. Lipids.

[17] Haines AP, Sanders TA, Imeson JD, Mahler RF, Martin J, Mistry M, Vickers M, Wallace PG (1986). Effects of a fish oil supplement on platelet function, haemostatic variables and albuminuria in insulin-dependent diabetics. Thromb Res.

[18] Stiefel P, Ruiz-Gutierrez V, Gajon E, Acosta D, Garcia-Donas MA, Madrazo J, Villar J, Carneado J (1999). Sodium transport kinetics, cell membrane lipid composition, neural conduction and metabolic control in type 1 diabetic patients. Changes after a low-dose n-3 fatty acid dietary intervention. Ann Nutr Metab.

[19] Altman DG, Bland JM (2005). Treatment allocation by minimisation. Bmj.

[20] Kulich KR, Madisch A, Pacini F, Piqué JM, Regula J, Van Rensburg CJ, Ujszászy L, Carlsson J, Halling K, Wiklund IK (2008). Reliability and validity of the Gastrointestinal Symptom Rating Scale (GSRS) and Quality of Life in Reflux and Dyspepsia (QOLRAD) questionnaire in dyspepsia: a six-country study. Health Qual Life Outcomes.

[21] Kohler A, Bittner D, Low A, von Schacky C (2010). Effects of a convenience drink fortified with n-3 fatty acids on the n-3 index. Br J Nutr.

[22] Akbari-Sedigh A, Asghari G, Yuzbashian E, Dehghan P, Imani H, Mirmiran P (2019). Association of dietary pattern with carotid intima media thickness among children with overweight or obesity. Diabetol Metab Syndr.

[23] Cocking S, Cable NT, Wilson MG, Green DJ, Thijssen DHJ, Jones H (2018). Conduit artery diameter during exercise is enhanced after local, but not remote. Ischemic Preconditioning. Front Physiol.

[24] Weiss EP, Fields DA, Mittendorfer B, Haverkort MA, Klein S (2008). Reproducibility of postprandial lipemia tests and validity of an abbreviated 4-hour test. Metabolism.

[25] Campbell MD, Walker M, Ajjan RA, Birch KM, Gonzalez JT, West DJ (2017). An additional bolus of rapid-acting insulin to normalise postprandial cardiovascular risk factors following a high-carbohydrate high-fat meal in patients with type 1 diabetes: a randomised controlled trial. Diab Vasc Dis Res.

[26] West DJ, Campbell MD, Gonzalez JT, Walker M, Stevenson EJ, Ahmed FW, Wijaya S, Shaw JA, Weaver JU (2015). The inflammation, vascular repair and injury responses to exercise in fit males with and without Type 1 diabetes: an observational study. Cardiovasc Diabetol.

[27] Faul F, Erdfelder E, Lang AG, Buchner A (2007). G*Power 3: a flexible statistical power analysis program for the social, behavioral, and biomedical sciences. Behav Res Methods.

[28] Woodman RJ, Mori TA, Burke V, Puddey IB, Barden A, Watts GF, Beilin LJ (2003). Effects of purified eicosapentaenoic acid and docosahexaenoic acid on platelet, fibrinolytic and vascular function in hypertensive type 2 diabetic patients. Atherosclerosis.

[29] Wong CY, Yiu KH, Li SW, Lee S, Tam S, Lau CP, Tse HF (2010). Fish-oil supplement has neutral effects on vascular and metabolic function but improves renal function in patients with Type 2 diabetes mellitus. Diabet Med.

[30] Stirban A, Nandrean S, Götting C, Tamler R, Pop A, Negrean M, Gawlowski T, Stratmann B, Tschoepe D (2010). Effects of n–3 fatty acids on macro- and microvascular function in subjects with type 2 diabetes mellitus. Am J Clin Nutr.

[31] Siniarski A, Haberka M, Mostowik M, Golebiowska-Wiatrak R, Poreba M, Malinowski KP, Gasior Z, Konduracka E, Nessler J, Gajos G (2018). Treatment with omega-3 polyunsaturated fatty acids does not improve endothelial function in patients with type 2 diabetes and very high cardiovascular risk: a randomized, double-blind, placebo-controlled study (Omega-FMD). Atherosclerosis.

[32] Lonn EM, Bosch J, Diaz R, Lopez-Jaramillo P, Ramachandran A, Hancu N, Hanefeld M, Krum H, Ryden L, Smith S (2013). Effect of insulin glargine and n-3FA on carotid intima-media thickness in people with dysglycemia at high risk for cardiovascular events: the glucose reduction and atherosclerosis continuing evaluation study (ORIGIN-GRACE). Diabetes Care.

[33] Turner D, Luzio S, Kilduff LP, Gray BJ, Dunseath G, Bain SC, Campbell MD, West DJ, Bracken RM (2014). Reductions in resistance exercise-induced hyperglycaemic episodes are associated with circulating interleukin-6 in type 1 diabetes. Diabet Med.

[34] Skrha J, Kalousova M, Svarcova J, Muravska A, Kvasnicka J, Landova L, Zima T, Skrha J (2012). Relationship of soluble RAGE and RAGE ligands HMGB1 and EN-RAGE to endothelial dysfunction in type 1 and type 2 diabetes mellitus. Exp Clin Endocrinol Diabetes.

[35] Galkina E, Ley K (2007). Vascular adhesion molecules in atherosclerosis. Arterioscler Thromb Vasc Biol.

[36] Clausen P, Jacobsen P, Rossing K, Jensen JS, Parving HH, Feldt-Rasmussen B (2000). Plasma concentrations of VCAM-1 and ICAM-1 are elevated in patients with Type 1 diabetes mellitus with microalbuminuria and overt nephropathy. Diabet Med.

[37] Chen H, Li D, Chen J, Roberts GJ, Saldeen T, Mehta JL (2003). EPA and DHA attenuate ox-LDL-induced expression of adhesion molecules in human coronary artery endothelial cells via protein kinase B pathway. J Mol Cell Cardiol.

[38] Ménégaut L, Jalil A, Thomas C, Masson D (2019). Macrophage fatty acid metabolism and atherosclerosis: the rise of PUFAs. Atherosclerosis.

[39] Darwesh AM, Sosnowski DK, Lee TY, Keshavarz-Bahaghighat H, Seubert JM (2019). Insights into the cardioprotective properties of n-3 PUFAs against ischemic heart disease via modulation of the innate immune system. Chem Biol Interact.

[40] Sheikh O, Vande Hei AG, Battisha A, Hammad T, Pham S, Chilton R (2019). Cardiovascular, electrophysiologic, and hematologic effects of omega-3 fatty acids beyond reducing hypertriglyceridemia: as it pertains to the recently published REDUCE-IT trial. Cardiovasc Diabetol.

[41] Poreba M, Rostoff P, Siniarski A, Mostowik M, Golebiowska-Wiatrak R, Nessler J, Undas A, Gajos G (2018). Relationship between polyunsaturated fatty acid composition in serum phospholipids, systemic low-grade inflammation, and glycemic control in patients with type 2 diabetes and atherosclerotic cardiovascular disease. Cardiovasc Diabetol.

[42] Mita T, Watada H, Ogihara T, Nomiyama T, Ogawa O, Kinoshita J, Shimizu T, Hirose T, Tanaka Y, Kawamori R (2007). Eicosapentaenoic acid reduces the progression of carotid intima-media thickness in patients with type 2 diabetes. Atherosclerosis.

[43] Hartweg J, Farmer AJ, Holman RR, Neil HA (2007). Meta-analysis of the effects of n-3 polyunsaturated fatty acids on haematological and thrombogenic factors in type 2 diabetes. Diabetologia.

[44] Bansal S, Buring JE, Rifai N, Mora S, Sacks FM, Ridker PM (2007). Fasting compared with nonfasting triglycerides and risk of cardiovascular events in women. JAMA.

[45] Langsted A, Nordestgaard BG (2019). Nonfasting versus fasting lipid profile for cardiovascular risk prediction. Pathology.

[46] Hartweg J, Perera R, Montori V, Dinneen S, Neil HA, Farmer A (2008). Omega-3 polyunsaturated fatty acids (PUFA) for type 2 diabetes mellitus. Cochrane Database Syst Rev.

[47] Leslie MA, Cohen DJA, Liddle DM, Robinson LE, Ma DWL (2015). A review of the effect of omega-3 polyunsaturated fatty acids on blood triacylglycerol levels in normolipidemic and borderline hyperlipidemic individuals. Lipids Health Dis.

[48] Khovidhunkit W, Kim MS, Memon RA, Shigenaga JK, Moser AH, Feingold KR, Grunfeld C (2004). Effects of infection and inflammation on lipid and lipoprotein metabolism: mechanisms and consequences to the host. J Lipid Res.

[49] Mansoori A, Sotoudeh G, Djalali M, Eshraghian MR, Keramatipour M, Nasli-Esfahani E, Shidfar F, Alvandi E, Toupchian O, Koohdani F (2015). Effect of DHA-rich fish oil on PPARgamma target genes related to lipid metabolism in type 2 diabetes: a randomized, double-blind, placebo-controlled clinical trial. J Clin Lipidol.

[50] Rivellese AA, Maffettone A, Iovine C, Di Marino L, Annuzzi G, Mancini M, Riccardi G (1996). Long-term effects of fish oil on insulin resistance and plasma lipoproteins in NIDDM patients with hypertriglyceridemia. Diabetes Care.

[51] Shidfar F, Keshavarz A, Hosseyni S, Ameri A, Yarahmadi S (2008). Effects of omega-3 fatty acid supplements on serum lipids, apolipoproteins and malondialdehyde in type 2 diabetes patients. East Mediterr Health J.

[52] Tenenbaum A, Fisman EZ (2018). Omega-3 polyunsaturated fatty acids supplementation in patients with diabetes and cardiovascular disease risk: does dose really matter?. Cardiovasc Diabetol.

[53] Crichton GE, Howe PRC, Buckley JD, Coates AM, Murphy KJ, Bryan J (2012). Long-term dietary intervention trials: critical issues and challenges. Trials.

[54] Julibert A, Bibiloni MDM, Tur JA (2019). Dietary fat intake and metabolic syndrome in adults: a systematic review. Nutr Metab Cardiovasc Dis.

[55] Li X, Bi X, Wang S, Zhang Z, Li F, Zhao AZ (2019). Therapeutic potential of ω-3 polyunsaturated fatty acids in human autoimmune diseases. Front Immunol.

