# Supplementary material for: Omega-3 polyunsaturated fatty acid supplementation versus placebo on vascular health, glycaemic control, and metabolic parameters in people with type 1 diabetes: a randomised controlled preliminary trial
Source: Cardiovasc Diabetol. 2020 Aug 12;19:127. doi: 10.1186/s12933-020-01094-5 (PMC7425064; doi:10.1186/s12933-020-01094-5)
Supplement: Supplementary file 1 — Additional file 1: Figure S1. Schematic representation of study design. n-3PUFA, omega-3 polyunsaturated fatty acid; = blood sampling; ultrasound imaging. Laboratory visits were scheduled at week 0, 12, 24, and 36. Table S1. Macronutrient composition of mixed-meal tolerance tests. Table S2. Erythrocyte fatty acid profiles in response to n-3PUFA supplementation or placebo in adults with type 1 diabetes. Mean ± SD. n=10 in each group. *, p<0.05; **, p<0.01; ***, p<0.001. n-3PUFA, omega-3 polyunsaturated fatty acids. All values are expressed as a percentage of total identified fatty acids after response factor correction. Table S3. Adjusted analysis for the effects of n-3PUFA supplementation on multiple health outcomes in adults with type 1 diabetes. Table S4. Pre-treatment demographic and clinical characteristics of intention-to-treat cohort. Table S5. Effect of omega-3 polyunsaturated fatty acids (n3PUFA) or placebo on cardiovascular and metabolic biomarkers, glycaemic parameters, measures of anthropometry, and vascular structure and function as per intention-to-treat analysis. [file 12933_2020_1094_MOESM1_ESM.docx]

**
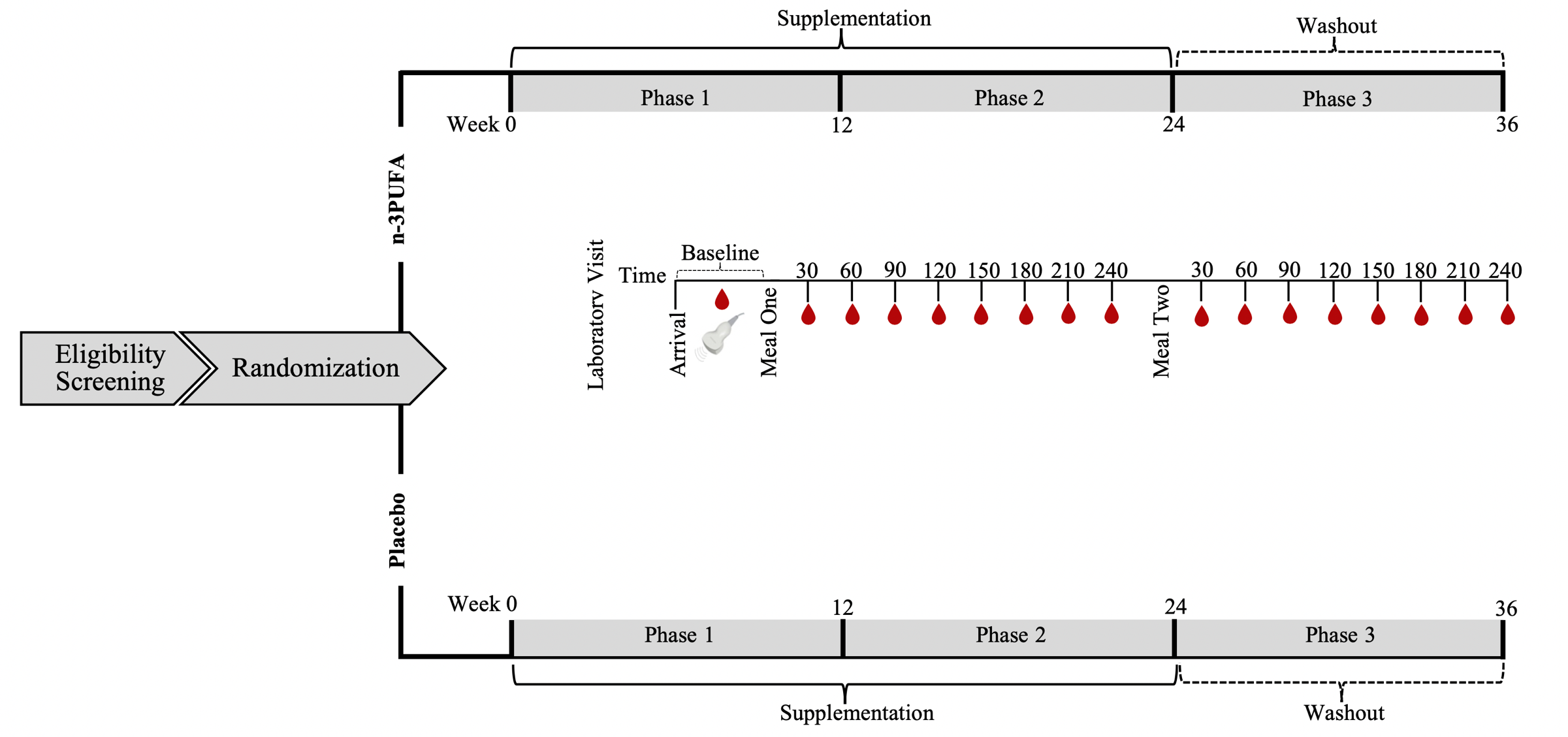
**


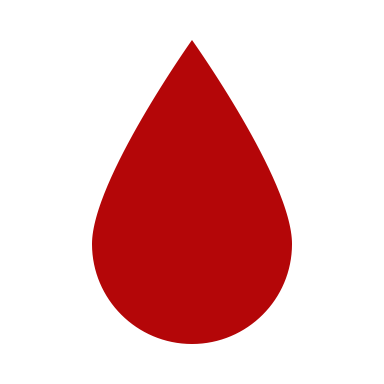


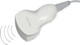
**Figure
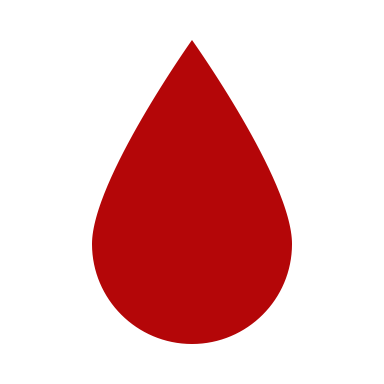

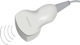
 S1.** Schematic representation of study design. n-3PUFA, omega-3 polyunsaturated fatty acid; = blood sampling; = ultrasound imaging. Laboratory visits were scheduled at week 0, 12, 24, and 36

|  | **Macronutrient composition (%)** | | | **Individualized carbohydrate content (g)** | | | | | | | | **Ingredients and manufacturer** |
| --- | --- | --- | --- | --- | --- | --- | --- | --- | --- | --- | --- | --- |
|  | CHO | FAT | PRO | Visit 1 | | Visit 2 | | Visit 3 | | Visit 4 | |  |
|  |  |  |  | n-3PUFA | Placebo | n-3PUFA | Placebo | n-3PUFA | Placebo | n-3PUFA | Placebo |  |
| **Meal 1** | 57 | 35 | 8 | 83±15 | 80±19 | 83±14 | 81±19 | 82±15 | 81±19 | 83±15 | 81±18 | Croissant (All Butter Croissant; Tesco, UK), butter (Lurpak^®^ Slightly Salted Spreadable; Arla Foods, Denmark) and Jam (Strawberry Jam Classic Recipe; Tesco, UK). |
| **Meal 2** | 54 | 27 | 19 |  |  |  |  |  |  |  |  | Basmati rice (Tesco, UK), chicken breast (Tesco, UK), a low-fat curry sauce (Tikka Masala Sauce; Tesco, UK) and clarified butter (Ghee; East End Foods, UK). |

**Table S1.** Macronutrient composition of mixed-meal tolerance tests

Note: The carbohydrate content of each meal was individualized (equalling 1 g of carbohydrate per kilogram of body mass). n-3PUFA, omega-3 polyunsaturated fatty acids.

| **Fatty Acid** | | **Pre-treatment** | | **3 months** | | **6 months** | | **9 months** | | p value |
| --- | --- | --- | --- | --- | --- | --- | --- | --- | --- | --- |
| Nomenclature | Name | n-3PUFA | Placebo | n-3PUFA | Placebo | n-3PUFA | Placebo | n-3PUFA | Placebo |  |
| **Saturated Fatty Acids** | |  |  |  |  |  |  |  |  |  |
| C14:0 | Myristic Acid | 0.33±0.13 | 0.31±0.11 | 0.25±0.77 | 0.29±0.08 | 0.34±0.12 | 0.33±0.11 | 0.28±0.07 | 0.34±0.10 | 0.389 |
| C16:0 | Palmitic Acid | 22.02±0.90 | 22.15±1.65 | 22.24±0.54 | 22.52±1.71 | 22.57±0.98 | 22.89±1.43 | 22.16±1.78 | 22.52±1.66 | 0.945 |
| C18:0 | Stearic Acid | 16.62±0.70 | 16.17±1.42 | 16.54±0.72 | 16.34±0.82 | 16.04±0.80 | 16.09±0.80 | 16.42±0.69 | 16.24±1.06 | 0.683 |
| C20:0 | Arachidic Acid | 0.22±0.03 | 0.20±0.03 | 0.21±0.02 | 0.21±0.03 | 0.20±0.03 | 0.19±0.03 | 0.21±0.03 | 0.21±0.02 | 0.637 |
| C22:0 | Behenic Acid | 0.50±0.15 | 0.47±0.11 | 0.45±0.11 | 0.60±0.10 | 0.51±0.12 | 0.49±0.05 | 0.52±0.12 | 0.55±0.08 | 0.077 |
| C24:0 | Lignoceric Acid | 1.28±0.20 | 1.16±0.29 | 1.29±0.22 | 1.42±0.21 | 1.22±0.16 | 1.23±0.25 | 1.22±0.23 | 1.21±0.12 | 0.340 |
| **Monounsaturated Fatty Acids** | |  |  |  |  |  |  |  |  |  |
| C16:1ω7 | Palmitoleic Acid | 0.26±0.17 | 0.36±0.20 | 0.21±0.09 | 0.29±0.13 | 0.24±0.11 | 0.36±0.14 | 0.24±0.09 | 0.34±0.21 | 0.940 |
| C18:1ω9 | Oleic Acid | 15.73±1.02 | 16.42±1.11 | 15.33±1.16 | 16.08±1.44 | 15.37±1.45 | 16.77±1.43 | 15.73±1.52 | 16.45±1.21 | 0.380 |
| C20:1ω9 | Gondoic Acid | 0.31±0.05 | 0.28±0.03 | 0.27±0.06 | 0.28±0.03 | 0.23±0.04 | 0.28±0.03 | 0.29±0.05 | 0.28±0.05 | 0.005** |
| C24:1ω9 | Nervonic Acid | 1.50±0.24 | 1.34±0.38 | 1.52±0.17 | 1.57±0.23 | 1.44±0.28 | 1.38±0.26 | 1.45±0.23 | 1.48±0.23 | 0.316 |
| **Omega-6 Polyunsaturated Fatty Acids** | |  |  |  |  |  |  |  |  |  |
| C18:2ω6 | Linoleic Acid | 11.74±1.22 | 13.18±3.06 | 11.09±1.46 | 11.81±1.41 | 12.17±2.37 | 11.90±1.59 | 11.80±1.40 | 11.79±1.60 | 0.271 |
| C18:3ω6 | γ-linolenic Acid | 0.07±0.05 | 0.06±0.03 | 0.04±0.03 | 0.04±0.02 | 0.08±0.07 | 0.06±0.03 | 0.04±0.03 | 0.07±0.04 | 0.237 |
| C20:2ω6 | Eicosadienoic Acid | 0.24±0.03 | 0.24±0.03 | 0.22±0.04 | 0.22±0.05 | 0.22±0.04 | 0.22±0.05 | 0.22±0.05 | 0.22±0.04 | 0.998 |
| C20:3ω6 | Dihomo-γ-linolenic Acid | 1.76±0.31 | 1.64±0.42 | 1.50±0.43 | 1.55±0.32 | 1.34±0.35 | 1.61±0.35 | 1.69±0.38 | 1.52±0.33 | 0.015** |
| C20:4ω6 | Arachidonic Acid | 15.24±1.14 | 14.58±2.00 | 13.79±1.10 | 15.05±1.09 | 12.81±1.19 | 14.63±1.34 | 14.97±0.92 | 15.04±1.50 | <0.001*** |
| C22:4ω6 | Adrenic Acid | 3.19±0.31 | 3.07±0.75 | 2.44±0.54 | 3.23±0.52 | 1.88±0.39 | 2.95±0.60 | 2.56±0.22 | 3.11±0.56 | <0.001*** |
| C22:5ω6 | Docosapentaenoic Acid | 074±0.15 | 0.69±0.18 | 0.50±0.23 | 0.65±0.22 | 0.45±0.14 | 0.59±0.16 | 0.56±0.11 | 0.66±0.12 | 0.076 |
| **Omega-3 Polyunsaturated Fatty Acids** | |  |  |  |  |  |  |  |  |  |
| C18:3ω3 | α-linolenic Acid | 0.19±0.05 | 0.18±0.06 | 0.16±0.04 | 0.15±0.04 | 0.17±0.07 | 0.17±0.05 | 0.15±0.03 | 0.18±0.04 | 0.347 |
| C20:5ω3 | Eicosapentaenoic Acid | 0.75±0.20 | 0.68±0.24 | 2.47±1.14 | 0.70±0.40 | 2.92±0.78 | 0.75±0.40 | 0.96±0.22 | 0.72±0.47 | <0.001*** |
| C22:5ω3 | Docosapentaenoic Acid | 2.62±0.31 | 2.55±0.51 | 3.70±0.78 | 2.61±0.26 | 3.79±0.72 | 2.59±0.36 | 3.01±0.50 | 2.60±0.37 | <0.001*** |
| C22:6ω3 | Docosahexaenoic Acid | 4.08±0.90 | 3.63±0.97 | 5.20±0.87 | 3.84±1.16 | 5.37±0.76 | 3.92±1.24 | 4.88±0.54 | 3.86±1.19 | 0.007** |
| ω3index | Omega-3 Index | 4.93±0.94 | 4.31±1.15 | 7.67±1.86 | 4.63±1.41 | 8.29±1.45 | 4.67±1.52 | 5.84±0.69 | 4.58±1.62 | <0.001*** |

**Table S2.** Erythrocyte fatty acid profiles in response to n-3PUFA supplementation or placebo in adults with type 1 diabetes

Mean ± SD. *n*=10 in each group. *, p<0.05; **, p<0.01; ***, p<0.001. n-3PUFA, omega-3 polyunsaturated fatty acids. All values are expressed as a percentage of total identified fatty acids after response factor correction.

| **Table S3.** Adjusted analysis for the effects of n-3PUFA supplementation on multiple health outcomes in adults with type 1 diabetes | | | | |
| --- | --- | --- | --- | --- |
| **Outcome** | **Model 1** | **Model 2** | **Model 3** | **Model 4** |
| **Vascular Health** |  |  |  |  |
| Vascular cell adhesion molecule-1 (ng/mL) | F = 0.387, p = 0.701, *ηp2* = 0.022 | F = 0.093, p = 0.934, *ηp2* = 0.007 | F = 0.627, p = 0.558, *ηp2* = 0.040 | F = 0.286, p = 0.835, *ηp2* = 0.022 |
| Intercellular adhesion molecule-1 (ng/mL) | F = 0.810, p = 0.435, *ηp2* = 0.045 | F = 1.447, p = 0.255, *ηp2* = 0.100 | F = 1.598, p = 0.221, *ηp2* = 0.096 | F = 1.101, p = 0.341, *ηp2* = 0.078 |
| Vascular endothelial growth factor (pg/mL) | F = 1.054, p = 0.349, *ηp2* = 0.058 | F = 2.203, p = 0.138, *ηp2* = 0.145 | F = 1.469, p = 0.247, *ηp2* = 0.089 | F = 0.980, p = 0.376, *ηp2* = 0.070 |
| E-Selectin (ng/mL) | F = 0.443, p = 0.723, *ηp2* = 0.025 | F = 0.428, p = 0.734, *ηp2* = 0.032 | F = 0.952, p = 0.424, *ηp2* = 0.060 | F = 0.550, p = 0.651, *ηp2* = 0.041 |
| P-Selectin (ng/mL) | F = 0.426, p = 0.735, *ηp2* = 0.024 | F = 2.319, p = 0.090, *ηp2* = 0.151 | F = 0.280, p = 0.839, *ηp2* = 0.018 | F = 0.705, p = 0.555, *ηp2* = 0.051 |
| Pentraxin-3 (ng/mL) | F = 0.752, p = 0.444, *ηp2* = 0.042 | F = 1.547, p = 0.237, *ηp2* = 0.106 | F = 1.143, p = 0.324, *ηp2* = 0.071 | F = 0.754, p = 0.440, *ηp2* = 0.055 |
| Tumor necrosis factor alpha (pg/mL) | F = 0.456, p = 0.650, *ηp2* = 0.026 | F = 1.041, p = 0.370, *ηp2* = 0.074 | F = 0.811, p = 0.468, *ηp2* = 0.051 | F = 0.381, p = 0.712, *ηp2* = 0.028 |
| Far wall carotid intima-media thickness (mm) | F = 1.205, p = 0.312, *ηp2* = 0.074 | F = 0.707, p = 0.490, *ηp2* = 0.056 | F = 1.031, p = 0.368, *ηp2* = 0.073 | F = 0.605, p = 0.549, *ηp2* = 0.052 |
| Flow mediated dilation (%) | F = 0.742, p = 0.486, *ηp2* = 0.042 | F = 1.009, p = 0.383, *ηp2* = 0.072 | F = 0.455, p = 0.624, *ηp2* = 0.029 | F = 0.462, p = 0.605, *ηp2* = 0.034 |
| Systolic blood pressure (mm/Hg) | F = 1.487, p = 0.238, *ηp2* = 0.080 | F = 1.969, p = 0.151, *ηp2* = 0.132 | F = 1.894, p = 0.169, *ηp2* = 0.112 | F = 1.954, p = 0.163, *ηp2* = 0.131 |
| Diastolic blood pressure (mm/Hg) | F = 0.440, p = 0.630, *ηp2* = 0.025 | F = 0.513, p = 0.586, *ηp2* = 0.038 | F = 0.514, p = 0.591, *ηp2* = 0.033 | F = 0.603, p = 0.555, *ηp2* = 0.044 |
| **Glycemic Parameters** |  |  |  |  |
| HbA1c (mmol/mol) | F = 0.450, p = 0.596, *ηp2* = 0.027 | F = 0.909, p = 0.342, *ηp2* = 0.070 | F = 0.413, p = 0.620, *ηp2* = 0.029 | F = 0.520, p = 0.578, *ηp2* = 0.041 |
| Fasting plasma glucose (mmol/L) | F = 0.864, p = 0.428, *ηp2* = 0.054 | F = 1.340, p = 0.282, *ηp2* = 0.109 | F = 1.162, p = 0.328, *ηp2* = 0.088 | F = 0.649, p = 0.529, *ηp2* = 0.056 |
| PPGR (mmol/L/min) | F = 2.058, p = 0.130, *ηp2* = 0.121 | F = 2.475, p = 0.092, *ηp2* = 0.184 | F = 2.416, p = 0.090, *ηp2* = 0.157 | F = 3.068, p = 0.048*, *ηp2* = 0.218 |
| **Metabolic Parameters** |  |  |  |  |
| Triglycerides (mmol/L) | F = 0.185, p = 0.867, *ηp2* = 0.015 | F = 0.412, p = 0.680, *ηp2* = 0.044 | F = 0.418, p = 0.686, *ηp2* = 0.040 | F = 0.332, p = 0.734, *ηp2* = 0.040 |
| PPTR (mmol/L/min) | F = 0.248, p = 0.823, *ηp2* = 0.016 | F = 0.062, p = 0.946, *ηp2* = 0.006 | F = 0.606, p = 0.582, *ηp2* = 0.045 | F = 0.511, p = 0.629, *ηp2* = 0.044 |
| Cholesterol (mmol/L) | F = 0.414, p = 0.668, *ηp2* = 0.036 | F = 2.290, p = 0.104, *ηp2* = 0.222 | F = 0.236, p = 0.788, *ηp2* = 0.026 | F = 0.235, p = 0.775, *ηp2* = 0.033 |
| Non-esterified fatty acids (mmol/L) | F = 1.164, p = 0.333, *ηp2* = 0.096 | F = 1.878, p = 0.188, *ηp2* = 0.190 | F = 0.890, p = 0.433, *ηp2* = 0.090 | F = 0.677, p = 0.520, *ηp2* = 0.088 |
| **Anthropometry** |  |  |  |  |
| Body weight (Kg) | F = 0.201, p = 0.851, *ηp2* = 0.012 | F = 0.052, p = 0.955, *ηp2* = 0.004 | F = 0.122, p = 0.914, *ηp2* = 0.008 | F = 0.128, p = 0.909, *ηp2* = 0.010 |
| Body mass index (kg/m^2^) | F = 0.530, p = 0.509, *ηp2* = 0.030 | F = 0.093, p = 0.821, *ηp2* = 0.007 | F = 1.281, p = 0.282, *ηp2* = 0.079 | F = 1.550, p = 0.237, *ηp2* = 0.107 |
| Body Fat (%) | F = 0.106, p = 0.837, *ηp2* = 0.006 | F = 0.100, p = 0.853, *ηp2* = 0.008 | F = 0.206, p = 0.745, *ηp2* = 0.014 | F = 0.371, p = 0.627, *ηp2* = 0.028 |

Mean ± SD. *n*=10 in each group. *, p<0.05; **, p<0.01; ***, p<0.001. PPGR, postprandial glucose response; PPTR, postprandial triglyceride response. Postprandial responses presented as area under the curve following two consecutive mixed-meal tolerance tests. Model 1: adjusted for pre-treatment n-3index only; Model 2: adjusted for pre-treatment n-3index, docosahexaenoic acid, eicosapentaenoic acid, docosapentaenoic acid, and alpha-linolenic acid; Model 3: adjusted for pre-treatment n-3index, age, and gender; Model 4: adjusted for pre-treatment n-3index, age, gender, age of diagnosis, duration of disease, and mode of insulin therapy.

|  | **n3PUFA** | **Placebo** |
| --- | --- | --- |
| *n* | 14 | 13 |
| Male | 10 | 9 |
| Female | 4 | 4 |
| Insulin therapy (*n*) |  |  |
| Multiple daily injection | 9 | 6 |
| Basal | 4^G^; 3^DG^; 2^D^ | 2^DG^; 2^G^; 1^D^; 1^I^ |
| Bolus | 7^A^; 2^L^ | 5^A^; 1^GL^ |
| CSII | 5^A^ | 7(4^A^; 3^L^) |
| Medication | 1 Atorvastatin  1 Lisinopril  1 Losartan | 1 Atorvastatin, Metformin, and Nefopam  1 Lansoprazole  1 Levothyroxine  1 Lisinopril and Pravastatin |
| Age (y) | 32±12 | 37±15 |
| Body Mass Index (kg/m^2^) | 25.94±4.14 | 27.68±5.62 |
| Duration of diabetes (y) | 15±13 | 23±12 |
| Age of diagnosis (y) | 17±7 | 13±7 |
| HbA1c (mmol/mol^-1^; %) | 56.78±12.41;7.30±3.30 | 64.23±14.70;8.00±3.50 |
| SBP (mm/Hg^-1^) | 130.00±10.38 | 131.62±10.95 |
| DBP (mm/Hg^-1^) | 78.00±8.58 | 76.31±5.86 |
| Omega-3 Index (%) | 5.14±1.04 | 4.56±1.15 |

**Table S4.** Pre-treatment demographic and clinical characteristics of intention-to-treat cohort.

Data presented as mean ± SD. CSII, continuous subcutaneous insulin infusion; HbA1c, glycated haemoglobin; SBP, systolic blood pressure; DBP, diastolic blood pressure. A, Aspart; D, Detemir; DG, Degludec; G, Glargine; GL, Glulisine; I, Isophane; L, Lispro.

**Table S5.** Effect of omega-3 polyunsaturated fatty acids (n3PUFA) or placebo on cardiovascular and metabolic biomarkers, glycaemic parameters, measures of anthropometry, and vascular structure and function as per intention-to-treat analysis.

| **Outcome** | **n-3PUFA (n)** | **Placebo (n)** | **Intention to treat analysis (last observation carried forward)^a^** |
| --- | --- | --- | --- |
| **Vascular Health** |  |  |  |
| Vascular cell adhesion molecule-1 (ng/mL) | 12 | 11 | F(_3, 63_) = 0.210, p = 0.889, ηp^2^ = 0.010 |
| Intercellular adhesion molecule-1 (ng/mL) | 12 | 11 | F(_1.80,37.69_) = 0.889, p = 0.410, ηp^2^ = 0.041 |
| Vascular endothelial growth factor (pg/mL) | 12 | 11 | F(_1.69, 35.46_) = 1.473, p = 0.243, ηp^2^ = 0.066 |
| E-Selectin (ng/mL) | 12 | 11 | F(_3, 63_) = 0.632, p = 0.597, ηp^2^ = 0.029 |
| P-Selectin (ng/mL) | 12 | 11 | F(_3, 63_) = 0.305, p = 0.822, ηp^2^ = 0.014 |
| Pentraxin-3 (ng/mL) | 12 | 11 | F(_1.54, 32.25_) = 0.670, p = 0.481, ηp^2^ = 0.031 |
| Tumour necrosis factor alpha (pg/mL) | 12 | 11 | F(_2.17, 45.51_) = 0.468, p = 0.645, ηp^2^ = 0.022 |
| Far wall CIMT (mm) | 13 | 11 | F (_1.83, 40.33_) = 1.393, p = 0.259, ηp^2^ = 0.060 |
| Flow-mediated dilation (%) | 12 | 11 | F (_2.21, 46.41_) = 0.622, p = 0.557, ηp^2^ = 0.029 |
| Systolic blood pressure (mm/Hg) | 14 | 13 | F(_3, 75_) =1.110, p = 0.350, ηp^2^ = 0.043 |
| Diastolic blood pressure (mm/Hg) | 14 | 13 | F(_1.86, 46.61_) = 1.066, p = 0.349, ηp^2^ = 0.041 |
| **Glycaemic Parameters** |  |  |  |
| Glycated haemoglobin (mmol/mol) | 14 | 13 | F(_1.82, 45.47_) = 0.730, p = 0.475, ηp^2^ = 0.028 |
| Fasting plasma glucose (mmol/L) | 13 | 11 | F(_3,66_) = 0.192, p = 0.902, ηp^2^ = 0.009 |
| PPGR (mmol/L/min) | 12 | 10 | F(_1.87, 37.39_) = 0.151, p = 0.847, ηp^2^ = 0.007 |
| **Metabolic Parameters** |  |  |  |
| Triglycerides (mmol/L) | 11 | 9 | F(_2.53,45.41_) = 0.138, p = 0.912, ηp^2^ = 0.008 |
| PPTR (mmol/L/min) | 11 | 9 | F(_2.43, 43.67_) = 0.236, p = 0.831, ηp^2^ = 0.013 |
| Cholesterol (mmol/L) | 11 | 9 | F(_1.96, 35.35_) = 0.399, p = 0.670, ηp^2^ = 0.022 |
| NEFA (mmol/L) | 11 | 9 | F(_2.43, 43.72_) = 0.943, p = 0.412, ηp^2^ = 0.050 |
| **Anthropometry** |  |  |  |
| Body weight (Kg) | 14 | 13 | F(_3,75_) = 0.981, p = 0.406, ηp^2^ = 0.038 |
| Body mass index (kg/m^2^) | 14 | 13 | F(_1.39, 34.85_) = 0.282, p = 0.676, ηp^2^ = 0.011 |
| Body fat (%) | 14 | 13 | F(_1.53, 38.25_) = 0.249, p = 0.721, ηp^2^ = 0.010 |

Table S6. CONSORT 2010 checklist

| Section/Topic | Item No | Checklist item | Reported on page No |
| --- | --- | --- | --- |
| Title and abstract | | | |
|  | 1a | Identification as a randomised trial in the title | 1 |
|  | 1b | Structured summary of trial design, methods, results, and conclusions (for specific guidance see CONSORT for abstracts) | 2-3 |
| Introduction | | | |
| Background and objectives | 2a | Scientific background and explanation of rationale | 4 |
|  | 2b | Specific objectives or hypotheses | 4 |
| Methods | | | |
| Trial design | 3a | Description of trial design (such as parallel, factorial) including allocation ratio | 5, 6 |
|  | 3b | Important changes to methods after trial commencement (such as eligibility criteria), with reasons | 5 |
| Participants | 4a | Eligibility criteria for participants | 5 |
|  | 4b | Settings and locations where the data were collected | 5 |
| Interventions | 5 | The interventions for each group with sufficient details to allow replication, including how and when they were actually administered | 6 |
| Outcomes | 6a | Completely defined pre-specified primary and secondary outcome measures, including how and when they were assessed | 9 |
|  | 6b | Any changes to trial outcomes after the trial commenced, with reasons | N/A |
| Sample size | 7a | How sample size was determined | 9 |
|  | 7b | When applicable, explanation of any interim analyses and stopping guidelines | N/A |
| Randomisation: |  |  |  |
| Sequence generation | 8a | Method used to generate the random allocation sequence | 6 |
|  | 8b | Type of randomisation; details of any restriction (such as blocking and block size) | 6 |
| Allocation concealment mechanism | 9 | Mechanism used to implement the random allocation sequence (such as sequentially numbered containers), describing any steps taken to conceal the sequence until interventions were assigned | 6 |
| Implementation | 10 | Who generated the random allocation sequence, who enrolled participants, and who assigned participants to interventions | 6 |
| Blinding | 11a | If done, who was blinded after assignment to interventions (for example, participants, care providers, those assessing outcomes) and how | 6 |
|  | 11b | If relevant, description of the similarity of interventions | 6 |
| Statistical methods | 12a | Statistical methods used to compare groups for primary and secondary outcomes | 9 |
|  | 12b | Methods for additional analyses, such as subgroup analyses and adjusted analyses | 9 |
| Results | | | |
| Participant flow (a diagram is strongly recommended) | 13a | For each group, the numbers of participants who were randomly assigned, received intended treatment, and were analysed for the primary outcome | Figure 1 |
|  | 13b | For each group, losses and exclusions after randomisation, together with reasons | Figure 1 |
| Recruitment | 14a | Dates defining the periods of recruitment and follow-up | 5 |
|  | 14b | Why the trial ended or was stopped | N/A |
| Baseline data | 15 | A table showing baseline demographic and clinical characteristics for each group | 23 |
| Numbers analysed | 16 | For each group, number of participants (denominator) included in each analysis and whether the analysis was by original assigned groups | 24 |
| Outcomes and estimation | 17a | For each primary and secondary outcome, results for each group, and the estimated effect size and its precision (such as 95% confidence interval) | 24 |
|  | 17b | For binary outcomes, presentation of both absolute and relative effect sizes is recommended | N/A |
| Ancillary analyses | 18 | Results of any other analyses performed, including subgroup analyses and adjusted analyses, distinguishing pre-specified from exploratory | Supplementary file |
| Harms | 19 | All important harms or unintended effects in each group (for specific guidance see CONSORT for harms) | 11 |
| Discussion | | | |
| Limitations | 20 | Trial limitations, addressing sources of potential bias, imprecision, and, if relevant, multiplicity of analyses | 14 |
| Generalisability | 21 | Generalisability (external validity, applicability) of the trial findings | 14 |
| Interpretation | 22 | Interpretation consistent with results, balancing benefits and harms, and considering other relevant evidence | 13-14 |
| Other information | | |  |
| Registration | 23 | Registration number and name of trial registry | 3 |
| Protocol | 24 | Where the full trial protocol can be accessed, if available | 3 |
| Funding | 25 | Sources of funding and other support (such as supply of drugs), role of funders | 3, 17 |
